# Development and validation of career sustainability scale for mid-career employees

**DOI:** 10.3389/fpsyg.2024.1442119

**Published:** 2024-12-27

**Authors:** Suyeon Kim, Heesu Lee, Sungmi Jin

**Affiliations:** ^1^Department of Human Resource Development, Chung-Ang University, Seoul, Republic of Korea; ^2^Department of Education, Chung-Ang University, Seoul, Republic of Korea

**Keywords:** mid-career, career sustainability, career development, scale development, validation

## Abstract

A sustainability perspective on careers builds a resilient career system by simultaneously considering individual’s current career needs and long-term career outcomes. The individual career agent’s strategy for achieving a sustainable career is a powerful approach to career development in an environment where an individual’s proactive career competencies are emphasized. Mid-career individuals, especially those facing career transitions, need to increase their sustainability by maintaining, renewing, and developing their current careers. To support sustainable career development for mid-career employees, a tool is needed to objectively diagnose the level of career sustainability, taking into account the characteristics of their career transitions. The purpose of this study is to develop and validate a mid-career employee career sustainability scale. The research methodology comprised four systematic scale development processes. First, an integrated literature review was conducted to develop a conceptual model of mid-career career sustainability. Second, an initial pool of career sustainability items was developed and subjected to expert content validation. Third, an exploratory factor analysis (EFA) was conducted on 257 participants to verify the reliability and validity of the preliminary items. Finally, a confirmatory factor analysis (CFA) was conducted on 534 participants to finalize the final items. The participants were all mid-career employees aged 40–55 currently employed in Korean organizations. The new scale reliably and validly measured mid-career career sustainability across four dimensions: meaning perception of career sustainability, skill acquisition for career sustainability, relationship building for career sustainability, environmental awareness for career sustainability.

## 1 Introduction

Career sustainability refers to the ability of individuals to adapt to changes in their career environment with sufficient economic security, and to have the opportunity to renew their careers according to their needs and interests (Greenhaus and Kossek, 2014). The concept of career sustainability emerged against the backdrop of key features of modern careers, such as increasing job insecurity, frequent organizational mobility, and rising unemployment (Rapuano, 2020). The Fourth Industrial Revolution, which began with the development of technology, has enabled technology to take over jobs that were previously performed by humans, resulting in many workers requiring higher job skills or losing their jobs (McDonald and Hite, 2018). In addition, employment flexibility, which has emerged as a result of an increasingly competitive business environment, has increased job polarization (Chin et al., 2019). This leads to job stress and employment insecurity, which threatens individuals’ mental and physical health (Keeley, 2015).

In a labor market that has become unstable due to rapidly changing business environments, individuals face realistic demands to pursue lifelong careers or lifelong employability rather than lifelong jobs (Toscanelli et al., 2019). In order to respond flexibly to the changing career landscape, efforts are needed to understand careers in different contexts (Rapuano, 2020). This emphasis on contextualizing career development has led to a movement to view careers through the lens of the concept of ‘sustainability’ (Ehnert and Harry, 2012; McDonald and Hite, 2018). Tong and Gao (2022) emphasized that career sustainability is an essential ability to maintain a current job or obtain a new job as needed. Indeed, at the core of career sustainability is an individual’s psychological response and interaction with social and contextual factors, which triggers a learning process in response to situations and events that require change and adaptation (Müller et al., 2022). Therefore, in this study, career sustainability is understood as a psychological process that involves reflecting on an individual’s motivations and values in relation to the external environment of the job and restructuring belief systems and competencies by shifting identities.

From a sustainability perspective, careers build resilient career systems by simultaneously considering current and future career needs (De Vos and Van der Heijden, 2017; Spurk et al., 2019). Individuals can achieve continuous career growth by securing opportunities for career renewal through a variety of work experiences (De Vos et al., 2020). Career sustainability can look different at various stages of career maturity, so it is important to consider the stage of career development of the individual. It is important to look at what changes occur in an individual’s career over time at various life stages (Savanevičienė et al., 2023). In particular, mid-career is a time of significant change, and the importance of career development for mid-career individuals is increasing (Seo and Kim, 2024). This period can lead to new career opportunities and further growth, but it can also lead to a period of stagnation (Hwang and Lee, 2015), so it is necessary to maintain and develop one’s career through career sustainability. In this study, we aim to identify the factors that enable mid-career professionals to continue their career development.

Despite the ongoing interest in career sustainability, the research is still in its infancy (Chin et al., 2022). Several researchers have proposed components of career sustainability, but the complex structure that forms them is not yet fully understood, so no consensus has yet been reached on how to measure career sustainability (Chin et al., 2019; Hallpike et al., 2022).

Research on the development of direct measures for career sustainability remains limited, with few studies available aside from Chin et al. (2022). The study by Chin et al. (2022) presents valuable empirical research aimed at measuring career sustainability; however, the scale developed did not account for career maturity, making it less suitable for mid-career employees. Given that career sustainability can vary depending on the stage of career maturity, measurement items need to be tailored to the relevant career development stages. Mid-career, in particular, is a critical period often associated with major career transitions, underscoring the increasing importance of career development at this stage (Chen and Waglay, 2024). Therefore, this study seeks to develop a valid scale for measuring career sustainability among mid-career employees. This process will be guided by a systematic scale development approach, including an integrative literature review, expert content review through a Delphi survey, and both preliminary and main surveys.

In order to improve the understanding of career sustainability and provide successful career development for mid-career professionals, it is necessary to develop a measurement tool that reflects the characteristics of mid-career professionals. By identifying the level of career sustainability of mid-career individuals based on a career sustainability diagnostic tool, individuals as career agents can compensate for their weak competencies and pursue career success, including career satisfaction and employment security. The career sustainability scale not only provides an objective indicator of sustainability, which is becoming increasingly important for many organizations, but also supports long-term sustainable talent management through the establishment of an integrated career development and management system.

## 2 Conceptualization of career sustainability

Career sustainability is the sequence of career experiences reflected through different patterns of continuity over time that provide meaning to individuals across different social spaces (Van der Heijden and De Vos, 2015). Career sustainability comprises psychological experiences across the lifespan that are shaped by multiple life domains such as work, social, and family. McDonald and Hite (2018) define career sustainability as ‘a range of work experiences that provide continuous growth and reproduction, bringing meaning and well-being to individuals over time and across multiple life contexts.’ Other definitions and characteristics of career sustainability described by various studies are as follows (Table 1).

**Table 1:** Definition of career sustainability from literature.

Author	Definition
Van der Heijden and De Vos (2015)	A sequence of an individual’s different career experiences, reflected through various patterns of continuity over time, across several social spaces, providing meaning to the individual.
McDonald and Hite (2018)	A variety of work experiences that provide continuous growth and renewal, which intersects multiple life contexts to bring about personal meaning and well-being over time.
Holling (2001)	A particular form of human sustainability, the capacity to create, test and maintain one’s adaptive capability.
Newman (2011)	Preserving and enhancing human capital, and restoring and maintaining balance.
Greenhaus and Kossek (2014)	Based on sufficient economic security, consistent with one’s career and values, adaptable to reflect changes as one’s needs and interests develop, renewability such that an individual has opportunities.
Kossek et al. (2014)	Having positive career experiences throughout the life in ways that promote organizational and individual efficacy.
Fleisher et al. (2015)	Increasing employees’ awareness for career capital acquisition, stimulating them to utilize resources, and having them reflect on how they can benefit from their job or future careers.

The conceptual framework for understanding and characterizing career sustainability that has been discussed to date can be expressed in terms of three dimensions: career actors, context and time (Van der Heijden and De Vos, 2015). Firstly, career actors. To enhance career sustainability, workers need to be proactive about their career sustainability. For individuals, this means creating career continuity through lifelong learning and active career management (Lawrence et al., 2017). The second dimension is context. The social space or context in which a career unfolds is another important characteristic of a sustainable career (De Vos et al., 2020). To better understand career sustainability, it is necessary to take a multi-stakeholder perspective (Colakoglu et al., 2006). Career sustainability works well when subsystems such as work, home, and community are interdependent and supported (Rapuano, 2020). The third dimension is time: career sustainability is complex, non-linear, and dynamic in nature (Nagy et al., 2019). The fact that individuals’ goals change over time complicates career planning and development (Skulmoski et al., 2021). Career-related decisions and events should be evaluated from a long-term perspective, as what appears to be a sustainable solution at one point in life may prove to be less sustainable in the long term (De Vos et al., 2020).

## 3 Scale development procedure

**Figure 1:**
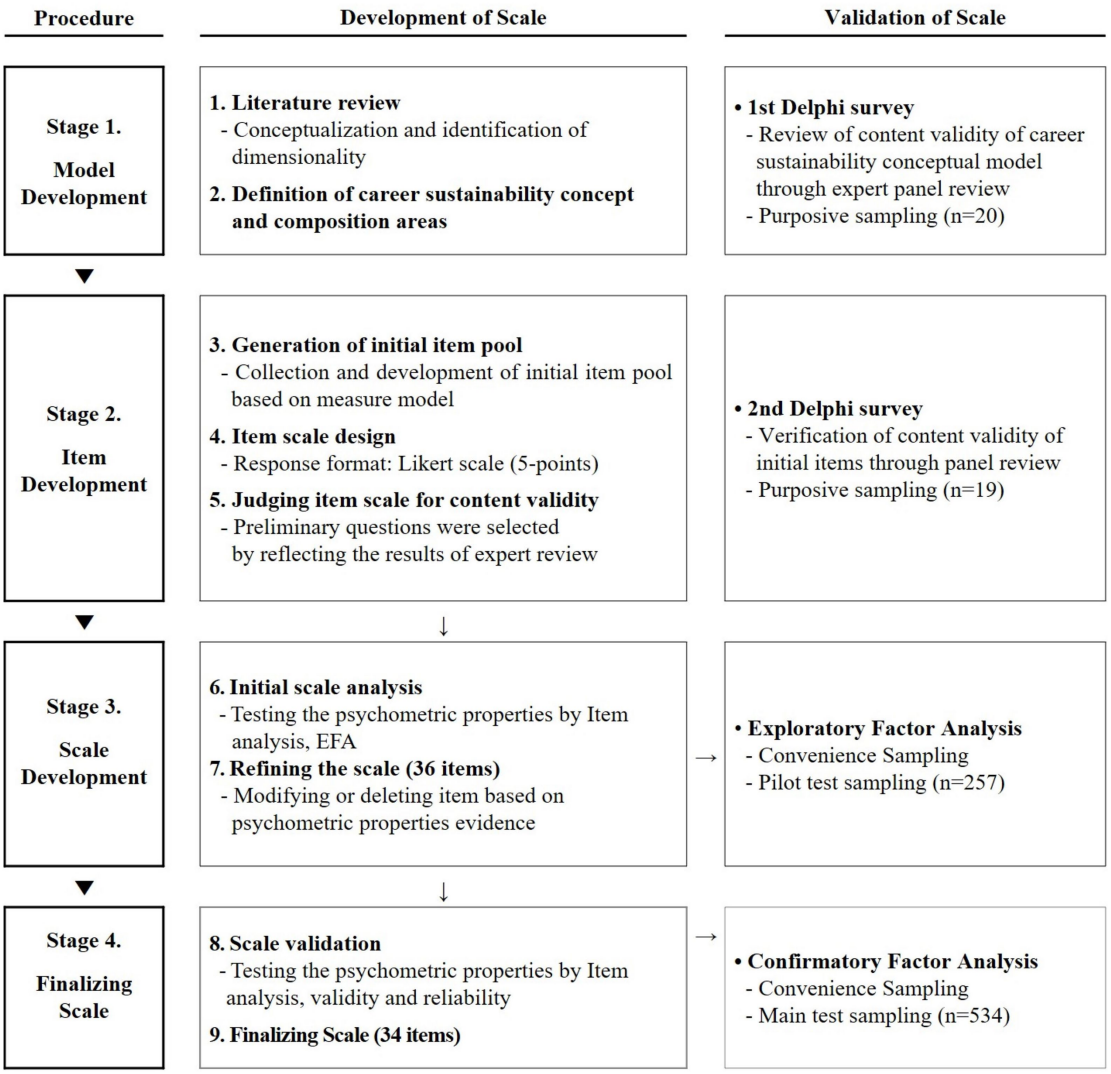
Scale development procedure.

In this study, we followed the step-by-step procedure suggested by Gerbing and Anderson (1988), Netemeyer et al. (2003) to develop a measure of career sustainability for mid-career employees. Study 1: Identified the multidimensional characteristics and sub-dimensions of mid-career career sustainability and developed a conceptual model of career sustainability that can serve as a measurement framework. Study 2: Generated measurement items that reflect the conceptual model of mid-career career sustainability developed in the previous step. Study 3: Secured the validity and reliability of the preliminary items through preliminary research and modified or removed inappropriate items. Study 4: Main research was conducted to ensure that the finalized and refined scale accurately measures the actual mid-career sustainability concept and constructs (Figure 1).

## 4 Results

### 4.1 Phase 1: Designing a career sustainability conceptual model

In this study, a conceptual model of career sustainability of mid-career employees was constructed through an integrated literature review. The conceptual model includes the definition, sub-factors, and measurement items of career sustainability. To construct the conceptual model, we adopted De Vos et al.'s (2020) sustainable career framework (individual, context, time) and Eby et al.’s (2003) career competences for career success framework (knowing why, knowing what, knowing whom) as guiding theories. The conceptual model was then confirmed through a first round of Delphi, an expert content validation procedure. First, the qualitative opinions of the expert panel and the characteristics of the research subjects were added to define career sustainability of mid-career professionals as follows. ‘Mid-career career sustainability is the ability to (1) reflect on whether one’s career so far is in line with one’s life values beyond the concept of the workplace, (2) cope/adapt to external environmental changes and social relationship changes that occur during mid-career, as well as internal conflicts about one’s position and behavior during life transitions, (3) develop one’s career through up-skilling and re-skilling, and (4) continuously create opportunities for career reproduction through professional relationship formation.’

The conceptual definition of each component area is as follows. First, meaning perception is the ability to realize one’s values and life identity through one’s career, and to set a career direction for this purpose; second, skill acquisition is the ability to demonstrate differentiated and competitive job skills based on continuous learning to sustain and develop one’s career in the future. Third, relationship building is defined as the ability to form various types of relationships in the course of an individual’s career, and to maintain and develop a professional network of collaboration and support in the context of career growth; and fourth, environmental awareness is defined as the ability to integrally understand the context of changing micro and macro environmental factors in one’s career field, and to reflect and develop them in one’s career.

### 4.2 Phase 2: Development of a scale for career sustainability

Next, preliminary questions were constructed based on the career sustainability sub-factors derived from the synthesis and analysis of existing career sustainability scales. In this study, three to five questions were derived for each factor to create preliminary questions. The preliminary questions for each factor were primarily based on the questions used in previous studies. For the development of items for mid-career career sustainability, the primary references were the career sustainability scales of Chin et al. (2022) and Kipkosgei (2019) (16 items and 6 items, respectively) and the sustainable career development scale of Argyropoulou (2021) (25 items). In addition, the results of scale development studies for each sub-factor were analyzed.

The questions developed in previous studies often consisted of general statements that did not take into account the target audience. Therefore, the meaning and wording of each item were modified to match the mid-career employees who were the subjects of this study. In addition, when the number of items in a sub-factor was insufficient compared to other factors, the researcher developed additional items to balance the constructs in the conceptual model. As a result of this process, a total of 70 items were obtained as a preliminary pool of items for measuring mid-career career sustainability, and a second Delphi survey was conducted to obtain a total of 58 preliminary items.

### 4.3 Phase 3: Exploratory factor analysis

A preliminary survey was conducted to validate the validity of the 58 items derived from the second Delphi survey and to improve the measurement items. The participants of the preliminary survey were limited to mid-career employees aged 40–55 years old working in office jobs in domestic companies. The sample of participants in the preliminary survey was obtained by applying a convenience sampling method. This study utilized an online method to ensure the convenience and anonymity of the respondents. Participants were recruited through job-specific bulletin boards in two online communities and two mobile communities where employees are actively participating, and the survey was conducted with their consent to collect personal information. The survey was conducted online or on mobile to ensure respondents’ convenience and anonymity. Of the 270 responses collected, 257 were used for the final analysis, excluding 13 responses from participants who were not eligible to participate (Table 2).

**Table 2:** Employee sample composition for pilot test (*n* = 257).

Category		Freq.	Rate	Category		Freq.	Rate
Gender	Male	113	44.0	Work Experience	10–15 years	86	33.5
	Female	144	56.0		16–20 years	108	42.0
Age	40–45	94	36.5		21–25 years	45	17.5
	46–50	104	40.5		26–30 years	16	6.2
	51–55	59	23.0		> 30 year	2	0.8
Degree	Under high School	23	8.9	Position	IT	50	19.5
	Junior College	66	25.7		Administration	73	28.4
	Bachelor	155	60.3		Marketing/Sales	55	21.4
	Master	11	4.3		R&D	37	14.4
	Doctor	2	0.8		Strategic	36	14.0
Employment Type	Full time	231	89.9		Others	6	2.3
	Temporary	26	10.1	Total		257	100

The data collected in the preliminary study was analyzed in the following order: item analysis, validity analysis, and reliability analysis (Gable and Wolf, 2012). The reliability and discriminant validity of the 58 items were checked through descriptive statistics, the correlation coefficient values for each item and the total correlation coefficient of the modified items. The means of each item ranged from 3.58 to 4.10, and all items had standard deviations of 0.150 or higher, which met the criteria. The maximum value of skewness was 0.86, and the maximum value of kurtosis was 1.32, both within the criteria. After reviewing each item and the overall correlation coefficient values, two items that did not meet the criteria (0.30 or less) were removed, leaving a total of 56 items for factor analysis.

Next, an exploratory factor analysis was conducted to determine whether the items comprising the mid-career career sustainability scale could be validly separated into sub-factors. The analysis revealed 20 items with factor loadings of less than 0.4. After a comprehensive review of the item content and criterion values, 20 items were excluded and a final factor analysis was conducted on 36 items. The Kaiser-Meyer-Olkin (KMO) values of the 36 items were reviewed to ensure that the sample for the factor analysis was adequate, and the KMO index for the preliminary sample was 0.941, which exceeded the adequacy criterion, indicating an adequate sample for factor analysis. Exploratory factor analysis showed that the 36 items were grouped into four factors with 55.557% variance explained. Both commonality and factor loadings were above 0.4.

Through item analysis and exploratory factor analysis, reliability analysis was conducted on a total of 36 items. After removing inappropriate items through item analysis and exploratory factor analysis, reliability analysis was conducted on a total of 36items using the coefficient of internal consistency. The overall reliability of the preliminary instrument was 0.949, which is a good level, and the reliability of each domain ranged from 0.890 to 0.946.

Based on this approach, we confirmed that the items of the scale were designed to measure diverse aspects of the construct. However, the internal consistency coefficient was somewhat high, raising concerns about potential item redundancy. To address this, we re-examined the multidimensionality identified in the exploratory factor analysis (EFA) conducted earlier. The results indicated that the items loaded onto four distinct factors, aligning with the theoretical structure of the construct set forth in this study. In addition to verifying internal consistency, we conducted further analyses to validate the construct, including content validity analysis with two doctoral-level experts in career development and discriminant validity testing in the main survey. Through this multifaceted approach, we ensured that the scale reflects the construct’s diverse aspects without overly relying on high internal consistency. This additional validation process was aimed at resolving the internal consistency issue and enhancing the robustness of the scale (Table 3).

**Table 3:** Factor structure of the domain items (EFA).

Factor	F1	F2	F3	F4	Cronbach’s α
Factor1: Skill acquisition for career sustainability					0.946
1. I periodically review my current competencies (knowledge, skills, attitudes) in order to continuously develop my career.	0.640	0.089	−0.045	0.036	
2. I try to upgrade my current skills to continuously develop my career.	0.613	−0.020	−0.142	0.082	
3. I try to learn the new skills to continuously develop my career.	0.698	0.117	−0.056	−0.067	
4. I can improve my quality of life through career development activities.	0.754	0.068	−0.052	−0.097	
5. I keep trying new tasks or experiences that are helpful to improve my expertise.	0.744	−0.014	0.078	0.108	
6. I try to figure out how the environment and trends surrounding my career are change and reflect them in my current way of working.	0.694	−0.040	−0.043	0.017	
7. In order to improve my work-related skills, I find and learn the necessary areas.	0.769	0.033	0.003	−0.010	
8. I continuously learn knowledge in my field of interest to develop my career.	0.775	0.010	0.004	0.030	
9. I am always aware of the latest technologies and research trends related to my job.	0.747	−0.040	−0.016	0.016	
10. I explore learning opportunities within and outside the organization to recognize and respond to changes in my job.	0.777	0.049	0.002	0.037	
11. I voluntarily participate in relevant education or training to develop the skills necessary for my career.	0.721	−0.050	−0.024	0.048	
12. I explore resources (human, material, and systemic) that can be used to improve my career.	0.731	0.000	0.039	0.047	
13. I can effectively utilize the resources necessary to achieve my career goals according to my work situation.	0.673	0.025	−0.005	0.082	
14. I accumulate scarce resources to achieve my career goals.	0.647	−0.106	0.012	0.138	
Factor2: Relationship building for career sustainability					0.891
1. I regularly meet with people who can help me in my career growth.	0.033	0.771	−0.044	−0.050	
2. I actively contact people who can help me achieve my career goals.	−0.127	0.809	−0.007	0.140	
3. I engage in professional gatherings to establish relationships with experts who can assist me in reaching my career objectives.	0.049	0.772	0.088	0.018	
4. I consistently share job-related information with experts in my field.	−0.032	0.749	−0.082	0.049	
5. I make new changes in my career based on job information obtained by interacting with people.	0.161	0.773	−0.012	−0.065	
Factor3: Meaning perception of career sustainability					0.890
1. I have a passion for my career to the extent that I can work tirelessly without realizing the passage of time.	−0.042	0.000	0.582	0.181	
2. I prioritize and dedicate time to building and sustaining career experiences over other activities.	0.128	−0.015	0.794	−0.165	
3. I believe that my career consists of tasks and activities that I enjoy and can continually immerse myself in.	−0.107	0.034	0.731	0.252	
4. I think my career contributes to ongoing growth and enhancement of expertise.	−0.099	0.102	0.700	0.123	
5. I believe that my career is contributing to external growth, such as increased income and status.	0.004	0.092	0.605	0.076	
6. I am aware of what I have and what I lack when comparing my current career to my goal career.	0.061	−0.090	0.617	0.138	
7. I have a clear career goal to continuously develop and maintain my career.	0.242	0.048	0.463	0.107	
8. Based on my past career experiences, I believe I can sustain my career in the future.	0.132	−0.008	0.797	−0.175	
Factor4: Environmental awareness for career sustainability					0.902
1. I try to make changes in my daily work to effectively achieve my career goals.	0.014	0.056	−0.031	0.670	
2. When performing tasks for new changes, I try to apply improved procedures.	−0.009	0.085	−0.071	0.587	
3. I voluntarily change my behavior in order to continuously develop my career.	0.121	0.013	−0.016	0.620	
4. I look for new ways to reinvent my career, even if old ways are easy.	0.095	−0.050	0.008	0.667	
5. I explore new career opportunities by examining the surrounding environment in changing situations.	0.218	0.029	0.066	0.577	
6. I explore various career alternatives with a deep interest in my future career.	0.147	−0.027	−0.028	0.634	
7. I trust myself and take responsibility for the career decisions I make.	−0.012	0.054	−0.015	0.636	
8. I can quickly adapt to changing career environments.	0.029	−0.009	−0.174	0.617	
9. I can flexibly adapt to changes in new ways of working according to changes in the career environment.	−0.004	0.007	0.028	0.737	
Eigenvalue	13.767	3.386	2.497	2.085	
Percentage of total variance	38.243	47.649	54.584	60.377	

### 4.4 Phase 4: Confirmatory factor analysis

The main study was conducted to provide final validation of the 36 items that had been tested for reliability and validity in the preliminary study. As in the preliminary study, the sample was collected through a convenience sampling method using occupation-specific bulletin boards in online and mobile communities of employees. The survey was administered online or via mobile. Of the 560 responses collected, 534 were finalized for analysis, excluding 26 non-responses from ineligible participants.

“Krejcie and Morgan (1970) suggested that, for statistical analysis, a sample size of 384 is adequate when the population exceeds 1,000,000, at a 95% confidence level. Crocker and Algina (1986) asserted that a sample size of 200 is sufficient to represent the population, while Gable and Wolf (2012) recommended a sample size that is four to five times the number of variables. Synthesizing these previous studies, a sample size of 400 or more was deemed appropriate.”

The data collected in the main study was analyzed in the same sequence as the preliminary study: item analysis, validity analysis, and reliability analysis (Gable and Wolf, 2012). The reliability and discriminant validity of the 36 items were verified through descriptive statistics, the correlation coefficients of each item, and the total correlation coefficient of the modified items. The means of each item ranged from 3.47 to 3.89, and all items had standard deviations of 0.150 or higher, meeting the criteria. The maximum skewness was 0.66, and the maximum kurtosis was 0.54, both within acceptable limits. After reviewing the correlation coefficients for each item and the overall items, two items that did not meet the criteria (values below 0.30) were removed, resulting in a total of 34 items used for confirmatory factor analysis (Table 4).

**Table 4:** Employee sample composition for main test (*n* = 534).

Category		Freq.	Rate	Category	Item	Freq.	Rate
Gender	Male	231	43.0	Work Experience	10–15 years	154	28.7
	Female	303	56.4		16–20 years	208	38.7
Age	40–45	234	43.6		21–25 years	82	15.3
	46–50	274	51.0		26–30 years	88	16.4
	51–55	26	4.8		> 30 year	2	0.4
Degree	Under high School	8	1.5	Position	IT	54	71.5
	Junior College	101	18.8		Administration	128	25.7
	Bachelor	373	69.5		Marketing/Sales	114	23.1
	Master	47	8.8		R&D	62	15.3
	Doctor	5	0.9		Strategic	55	14.2
Employment Type	Full time	497	92.6		Others	20	4.3
	Temporary	37	6.9	Total		534	100

**Figure 2:**
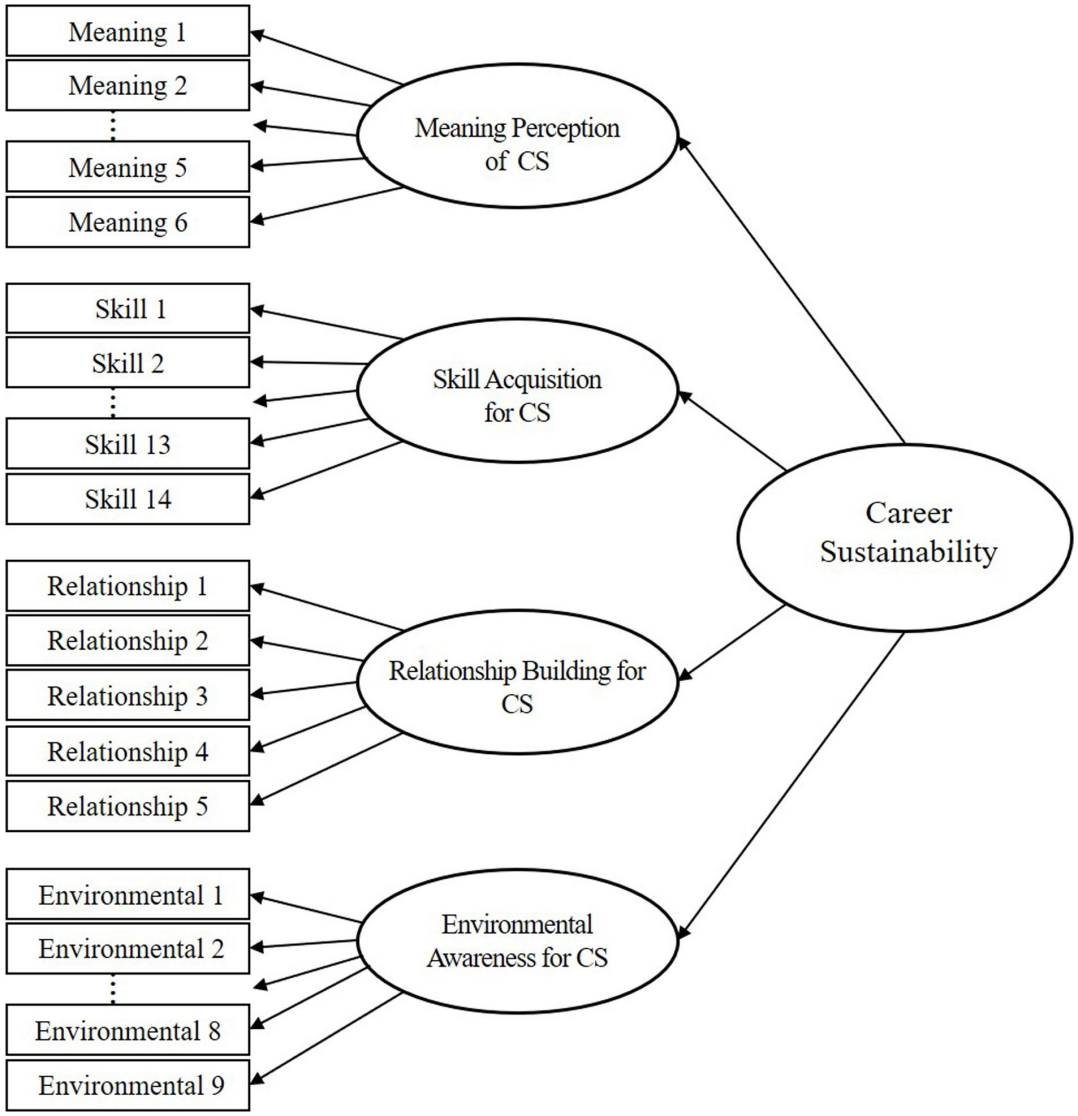
The second-order model of the factor structure of the career sustainability.

To verify the discriminant validity of the mid-career career sustainability scale and to review the final factor structure, a confirmatory factor analysis (CFA) was conducted. The AMOS 22.0 program was used for the confirmatory factor analysis. In this study, career sustainability was constructed as a multidimensional sub-factor scale. To this end, career sustainability was constructed as a multidimensional sub-scale of meaning perception of career sustainability, skill acquisition for career sustainability, relationship building for career sustainability, and environmental awareness for career sustainability, and a secondary hierarchical model was established based on this. The results of the model fit analysis showed that the TLI 0.960, CFI 0.963, RMSEA 0.037, and SRMR 0.032 were adequate for all fit indicators (Figure 2; Table 5).

**Table 5:** The model fit indices for confirmatory factor model of career sustainability.

	*χ* ^2^	df	RMSEA (90% CI)	CFI	TLI	SRMR
2nd order model	898.911	521	0.037	0.963	0.960	0.032

RMSEA, root mean square error of approximation; CFI, comparative fit index; TLI, Tucker-Lewis index; SRMR, standardized root mean square residual.

Next, discriminant validity was checked between the subscales of the mid-career career sustainability scale. Discriminant validity was further tested using correlation coefficients (r) and confidence intervals using standard errors (S.E.). As a result of the analysis, all correlation coefficient values did not contain 1 within ±2 S.E., so discriminant validity was secured. The discriminant validity of the sub-scales of the Career Sustainability Scale has been completed. Based on the results of the analyses, it was found that each of the subscales of career sustainability - meaning perception, skill acquisition, relationship building, and environmental awareness - reflected unique content.

To verify the convergent validity of the mid-career career sustainability scale, we checked the standardized factor loadings (0.5 or higher), concept reliability (0.7 or higher), and average variance extracted (AVE, 05 or higher). First, the standardized coefficients of each item were examined and found to be significant at *p* < 0.001, ranging from 0.599 to 0.750 for meaning perception, 0.653 to 0.758 for skill acquisition, 0.741 to 0.813 for relationship building, and 0.663 to 0.753 for environmental awareness. The average variance extracted (AVE) values were 0.521 for meaning perception, 0.576 for skill acquisition, 0.612 for relationship building, and 0.572 for environmental awareness, all of which exceeded the threshold of 0.5. Finally, the conceptual reliability values were 0.867 for meaning perception, 0.950 for skill acquisition, 0.887 for relationship building, and 0.923 for environmental awareness, all of which met the criterion (0.7 or higher). Based on the above three criteria (standardization coefficient, AVE, and concept reliability), convergent validity was determined to exist in all domains of career sustainability (Table 6).

**Table 6:** Confirmatory factor analysis of the career sustainability items.

Factor	Item	*β*	SE	*t*	AVE	Cronbach’s α
Meaning perception	1. I prioritize and dedicate time to building and sustaining career experiences over other activities.	0.699			0.521	0.845
	2. I believe that my career consists of tasks and activities that I enjoy and can continually immerse myself in.	0.699	0.073	15.614		
	3. I think my career contributes to ongoing growth and enhancement of expertise.	0.75	0.067	12.686		
	4. I believe that my career is contributing to external growth, such as increased income and status.	0.599	0.074	14.652		
	5. I have a clear career goal to continuously develop and maintain my career.	0.692	0.07	14.527		
	6. Based on my past career experiences, I believe I can sustain my career in the future.	0.699	0.071	14.655		
Skill acquisition	1. I periodically review my current competencies (knowledge, skills, attitudes) in order to continuously develop my career.	0.714			0.576	0.938
	2. I try to upgrade my current skills to continuously develop my career.	0.702	0.065	15.931		
	3. I try to learn the new skills to continuously develop my career.	0.729	0.067	16.533		
	4. I can improve my quality of life through career development activities.	0.653	0.061	14.808		
	5. I keep trying new tasks or experiences that are helpful to improve my expertise.	0.715	0.065	16.211		
	6. I try to figure out how the environment and trends surrounding my career are change and reflect them in my current way of working.	0.739	0.06	16.783		
	7. In order to improve my work-related skills, I find and learn the necessary areas.	0.758	0.06	17.208		
	8. I continuously learn knowledge in my field of interest to develop my career.	0.751	0.064	17.048		
	9. I am always aware of the latest technologies and research trends related to my job.	0.756	0.067	17.163		
	10. I explore learning opportunities within and outside the organization to recognize and respond to changes in my job.	0.703	0.062	15.943		
	11. I voluntarily participate in relevant education or training to develop the skills necessary for my career.	0.742	0.065	16.855		
	12. I explore resources (human, material, and system) that can be used to improve my career.	0.734	0.06	16.66		
	13. I can effectively utilize the resources necessary to achieve my career goals according to my work situation.	0.668	0.058	15.133		
	14. I accumulate scarce resources to achieve my career goals.	0.724	0.063	16.436		
Relationship building	1. I regularly meet with people who can help me in my career growth.	0.789			0.612	0.886
	2. I actively contact people who can help me achieve my career goals.	0.813	0.06	17.191		
	3. I engage in professional gatherings to establish relationships with experts who can assist me in reaching my career objectives.	0.804	0.062	18.805		
	4. I consistently share job-related information with experts in my field.	0.741	0.058	19.039		
	5. I make new changes in my career based on job information obtained by interacting with people.	0.754	0.057	18.436		
Environmental awareness	1. I try to make changes in my daily work to effectively achieve my career goals.	0.723			0.572	0.902
	2. When performing tasks for new changes, I try to apply improved procedures.	0.682	0.059	15.378		
	3. I voluntarily change my behavior in order to continuously develop my career.	0.738	0.059	16.679		
	4. I look for new ways to reinvent my career, even if old ways are easy.	0.744	0.065	16.823		
	5. I explore new career opportunities by examining the surrounding environment in changing situations.	0.753	0.06	17.039		
	6. I explore various career alternatives with a deep interest in my future career.	0.742	0.064	16.779		
	7. I trust myself and take responsibility for the career decisions I make.	0.678	0.06	15.301		
	8. I can quickly adapt to changing career environments.	0.663	0.058	14.938		
	9. I can flexibly adapt to changes in new ways of working according to changes in the career environment.	0.668	0.057	15.062		

Reliability validation was conducted to finalize the mid-career career sustainability scale. The validation method was checked by deriving item-total correlation coefficients for each subfactor, reliability coefficients when removing items, overall reliability coefficients for each factor, and reliability coefficients for the entire career sustainability scale. First, the Cronbach’s *α* value for the entire career sustainability scale was 0.962, which is high. Next, we analyzed the reliability coefficients for each sub-factor, and found that the reliability coefficients for meaning perception 0.845, skill acquisition 0.938, relationship building 0.886, and environmental awareness 0.902 were high. We also checked whether the Cronbach’s α values increased when items were removed and found that none of the items reduced internal consistency. This confirms the reliability of the mid-career career sustainability scale.

The Mid-career Career Sustainability Scale developed and validated in this study (see Table 6) consists of 34 items, including 6 items on meaning perception, 14 items on skill acquisition, 5 items on relationship building, and 9 items on environmental awareness.

## 5 Discussion and conclusion

The main objective of this study is to develop and validate a scale to measure career sustainability of mid-career employees. To address this task, a theory and conceptual model of career sustainability was developed through an integrative literature review, and a pool of preliminary measurement items of career sustainability was developed. The conceptual model and preliminary measurement items were reviewed for appropriateness through a Delphi survey, and 58 preliminary measurement items were obtained. The validity and reliability of the developed preliminary measures were verified through a preliminary survey of mid-career employees aged 40–55 working in Korean companies. Based on the results of 257 responses, an exploratory factor analysis was conducted to determine the number of factors and item composition. In the main survey, the reliability and validity of the measurement model and items were confirmed to be at an appropriate level through confirmatory factor analysis based on the results of 534 responses of mid-career employees. The mid-career career sustainability model established through this study was found to include the multidimensional dimensions of career sustainability.

The components of mid-career career sustainability developed in this study are meaning perception, skill acquisition, relationship building, and environmental awareness, respectively. Meaning perception is defined as the ability to realize one’s values and life identity through one’s career and to set a career direction for this purpose. It is divided into career commitment, career satisfaction, career goals, and career future orientation. Skill acquisition is related to professional knowledge and skills that individuals acquire in the process of preparing and experiencing their careers. This is defined as the ability to demonstrate differentiated and competitive job skills based on continuous learning to continuously advance one’s career. The dimensions were divided into career reproduction, knowledge and skills learning, and resource utilization.

Relationship building is defined as the ability of individuals to form various types of relationships over the course of their careers and to maintain and develop professional networks of co-operation and support in the context of career growth. A single factor, career networking, was used as the measure of career continuity relationship building. Environmental awareness is defined as the ability to integrate the changing context of micro and macro environmental factors in one’s career field to reflect and develop one’s career. It is comprised of situational and contextual awareness, change orientation, and career adaptability.

The mid-career career sustainability scale developed in this study is significant in that it consists of individual, context, and time dimensions, allowing for a multidimensional study that encompasses organizational and social contexts. This supports De Vos et al.'s (2020) theory that career sustainability is a multidimensional and simultaneous phenomenon that is shaped by the individual, their relationships with others, and their social context.

Meaning perception, skill acquisition, and relationship building are regarded as components at the individual level from a career competency perspective (Jin, 2013; DeFillippi and Arthur, 1994), though they also relate to the temporal aspect in terms of midlife development (Rapuano, 2020). Additionally, environmental awareness can be explored within the contextual domain (Colakoglu et al., 2006). This study aims to concretize the concept of career competencies introduced as essential abilities for individuals to develop career sustainability at the individual level into behavioral patterns.

At the individual level, mid-career employees can realize their values through accumulated career experiences, enabling them to establish a clear life identity and set future career directions (Van der Heijden et al., 2024). To sustain and further develop their careers, they acquire essential skills and knowledge (Newman, 2011) and build diverse interpersonal relationships that expand their career networks (Greenhaus and Kossek, 2014).

From a contextual perspective, this study adopts a macro-level view of the changing labor market context. In today’s labor market, technological advancement is driving significant shifts in traditional jobs, particularly disadvantaging mid-career employees who may struggle to keep up with these advances (Chen and Waglay, 2024). As technological changes create entirely new skills and job opportunities, impacting the labor market (Pandya and Wang, 2024), it is essential to understand the varied career contexts surrounding individuals and to seek ways to sustain and continuously develop their careers.

From a temporal perspective, this study incorporates a life-span development approach, identifying mid-career tasks and proposing career sustainability as a way to navigate these challenges (Chin et al., 2022; Sadler, 2000). Time is not an isolated aspect but is organically connected to the individual and contextual dimensions. In the career sustainability framework for mid-career employees, the temporal dimension serves as a critical link among the three aspects and is integrated into the study’s design.

Although an individual’s career is significantly influenced by changes in organizational and social environmental contexts, studies on developing career sustainability scales have focused on psychological or behavioral aspects as an extension of an individual’s subjective career success. Based on the Career Sustainability Scale, mid-career professionals are expected to be able to objectively assess their level of career sustainability at the individual, contextual and temporal levels, and to compensate for their weaknesses.

In addition, we secured the objectivity of the career sustainability scale by utilizing a systematic scale development process. Previous studies on career sustainability scales had limitations in that they relied on the subjective opinions of a few experts in the content validation process, or the scales were not developed through a systematic process, such as interviewing a small number of people to construct the items without separate validation (Argyropoulou, 2021; Chin et al., 2022; Kipkosgei, 2019). In this study, the conceptual model was derived by selecting and reviewing relevant data based on objective criteria through an integrative literature review.

In the workplace, the level of career sustainability of mid-career workers can provide a basis for determining which career development programs to develop and deliver. For mid-career workers with low levels of perceived career sustainability, support activities can be provided to help them make meaningful sense of their career experiences and prepare for their future careers, and for mid-career workers with low levels of skills acquisition for career sustainability, vocational training programs can be designed to help them update their careers (Chen and Waglay, 2024). Mid-career workers who have difficulty networking for career advancement can be coached in career redesign through consultation with a career specialist (Greenhaus et al., 2024). Finally, mid-career workers who are less aware of changes in their career environment can be provided with information about changes in the overall industry and career environment. As such, this study provides a substantive understanding of the career sustainability content of mid-career workers, which can be used as a useful basis for exploring tailored strategies to enhance mid-career workers’ career sustainability.

## 6 Limitation and suggestion

The limitations of this study and suggestions for future research are as follows. First, post-validation should be conducted to stabilize the developed scale. In order to validate the validity of career sustainability scales, the post-validation process should be conducted continuously from the time the tool is developed. In this study, the validity of the career sustainability measurement tool developed in the form of a scale was verified by checking the convergent validity and discriminant validity. In further research, the validity of the developed scale should be continuously validated using various methods in parallel to enhance the usability and stability of the new scale. The study by Chin et al. (2022) provided preliminary evidence for construct validity by examining the causal relationships between the developed career sustainability scale and variables such as career plateau, career satisfaction, and psychological well-being. Similarly, this study’s findings will allow us to predict factors influencing career sustainability among mid-career professionals and to test the predictive validity of the scale developed in this study.

Second, although the career sustainability scale developed in this study was aimed at mid-career employees in the corporate world, the final questionnaire did not capture the characteristics of mid-career employees. For example, career stagnation and struggles to maintain productivity, which are characteristic of mid-career, were not reflected in the final questionnaire. Even so, the findings of this study are meaningful from the perspective of career maintenance among mid-career employees. The career sustainability scale developed in this study can serve as an important indicator for developing and renewing their careers. Future research should provide direction on how to make the career development tasks of mid-career workers more explicit in the Delphi process, so that the career sustainability scale for mid-career workers is better characterized.

Third, in order to provide as objective a measure of an individual’s preparedness for career sustainability as possible, this study has endeavored to ensure sufficient content review and scientific rigor. However, the measurement tool developed is a self-reported scale, and as a result, it has limitations in measuring mid-career professionals’ perceptions of each area of career sustainability. The self-reported scale has limitations due to potential bias arising from subjectivity; however, it also offers advantages, such as broad applicability and the ability to measure a wide range of psychological characteristics (e.g., career satisfaction, career identity), thereby extending the scope of research. Additionally, because the survey format is simple and responses are easy to provide, many participants can readily engage, thus enhancing the applicability of the research. In future studies, to obtain a clearer measurement of career sustainability, it is necessary to comprehensively consider various indices and indicators that can be used to determine the level of career sustainability along with the measurement scale developed in this study.

## Data Availability

The raw data supporting the conclusions of this article will be made available by the authors, without undue reservation.
